# Young patients and gastrointestinal (GI) tract malignancies - are we addressing the unmet needs?

**DOI:** 10.1186/s12885-016-2676-4

**Published:** 2016-08-12

**Authors:** G. Perl, S. Nordheimer, S. Lando, C. Benedict, B. Brenner, S. Perry, G. Shmoisman, O. Purim, L. Amit, S. M. Stemmer, I. Ben-Aharon

**Affiliations:** 1 Institute of Oncology, Davidoff Center, Rabin Medical Center, Kaplan St, Petah-Tiqva, 49100 Israel; 2 Sackler Faculty of Medicine, Tel-Aviv University, Tel Aviv, Israel; 3 Department of Psychiatry & Behavioral Sciences, Memorial Sloan Kettering Cancer Center, New York, NY USA

**Keywords:** Gastrointestinal (GI) cancer, Survivorship, Unmet needs

## Abstract

**Background:**

Recent epidemiological studies indicate the rate of gastrointestinal (GI) malignancies among younger patients is increasing, mainly due to colorectal cancer. There is a paucity of data regarding the magnitude of treatment-related symptoms, psychosocial issues and potential unmet needs in this population. We aimed to characterize the needs of this population to evaluate whether unmet needs could be targeted by potential intervention.

**Methods:**

Female and male patients diagnosed with cancer of the gastrointestinal tract <40y retrospectively completed a questionnaire to evaluate symptoms, daily function and unmet needs at pre-treatment, during and post-treatment. Comparisons were made by gender, disease stage and treatment modality. Multiple linear regression models evaluated effects of demographics, symptoms and needs on multiple domains of health-related-quality-of-life (using Short-Form Health Survey-12 and CARES).

**Results:**

Fifty patients were enrolled (52 % female) to a pilot study. Median age at diagnosis was 35.5y (range, 21-40y). The symptoms that significantly increased from baseline to during and post-treatment were: diarrhea (37 %), sleeping disorder (32 %) and sexual dysfunction (40 %). Patients also reported significant deterioration in occupational activities and coping with children compared with baseline. Female patients reported significant unmet need for nutritional counseling and psychosocial support compared to male patients (*p* < 0.05). Patients treated with multimodality-treatment presented higher rates of unmet needs (*p* = 0.03).

**Conclusions:**

Young patients with GI cancers represent a group with unique characteristics and needs compared with published evidence on other young-onset malignancies. The distinctive symptoms and areas of treatment-related functional impairments indicate there are unmet needs, especially in the area of psychosocial support and nutritional counseling.

## Background

Young adults (YA) with gastrointestinal malignancies comprise a unique group that may be underrepresented among general YA cancer patient populations. The National Surveillance, Epidemiology, and End Results (SEER) registry data indicates that more than 140,000 people are diagnosed annually with colorectal cancer (CRC) in the US, and approximately half of them are women. Of the 70,480 women diagnosed with CRC in 2010, 3 to 5 % were younger than the age of 40. Incidence rates of CRC in young women (age 20–49) increased 1.6 % per year and up to 5.6 % per year in women aged 20 to 29 years from 1999 to 2005 [1–3]. The rate of other young-onset GI malignancies such as gastric or esophageal cancer is significantly lower, yet not rare. While overall gastric cancer incidence has steadily declined in many countries over the past 50 years, gastroesophageal rates are generally increasing in the western world particularly among YA populations. A recent study demonstrated that the incidence rate for noncardia gastric cancer declined among all race and age groups except for whites aged 25 to 39 years, for whom it had increased [4]. Recent studies characterizing YA patients with early-onset gastrointestinal malignancies have indicated that younger patients experience worse adverse effects of therapy (e.g., nausea and vomiting) compared with older patients [5, 6]. The magnitude of symptoms in the population of YAs with GI malignancies remains to be elucidated.

YA cancer patients often face unique challenges, including treatment-related infertility, interruption of academic and professional activities, and responsibilities for young children [7]. Decreased energy and sexual drive and the associated strains on relationships have been identified as substantial stressors in mixed-age survivors [8]. Among adolescent and young adult (AYA) survivors with mixed cancers, the results portrayed a unique spectrum of psychosocial symptom and subsequent burden. A recent study compared YAs with breast cancer to those with CRC and demonstrated a differential pattern of symptom burden and symptom severity, whereas CRC patients experienced worse symptoms [6].

It is well established that YAs with cancer often have high rates of unmet service and supportive care needs [7–11], which are associated with decrements in quality of life (QOL) [8]. Limited work has focused on YA CRC patients, however, and the unique challenges CRC survivors face are not well understood. Further work is needed to characterize the unmet needs of YA CRC patients, specifically, in order to develop targeted interventions that address specific areas of difficulty and improve overall quality of life.

Despite limited research in CRC, substantial evidence in other YA cancer populations indicates high rates of psychosocial difficulties and stressors. For example, younger women with breast cancer are at higher risk of distress throughout the disease and treatment spectrum [12–16] and more often face psychosocial challenges including treatment-related infertility, interruption of daily occupation, coping with spouse and young children, and decreased energy and sex drive [16]. Due to the paucity of data regarding the physical and psychosocial symptoms and needs of young patients with GI cancers [6, 17, 18], our study aimed to characterize CRC patients’ specific needs and quality of life concerns. This information may be used to identify potential targets of intervention for this unique population.

## Methods

### Study participants

The study cohort was comprised of patients diagnosed with cancer of the gastrointestinal tract (esophagus, gastric, colon, rectum, anal) between 6 months and two years prior to enrollment. Patients were eligible if they were: younger than 40 years old at diagnosis; had a Karnofsky Performance Status of 80 or above or an Eastern Cooperative Oncology Group (ECOG) score of 0 or 1.

### Procedures

This was a cross-sectional, retrospective survey. Participants were identified from the Davidoff center patient database and approached by the study team for a designated visit or a phone call. Following completion of informed consent, the patient completed a self-report questionnaire. The patient survey assessed demographic characteristics, reproductive factors (e.g., menstruation and pregnancy history); symptoms and health-related quality of life issues including psychosocial and physical functioning domains; potential barriers to patient care and the quality of health care received. The survey was modified to refer to three separate time periods: baseline or pre-treatment (T1), during treatment (T2), and post-treatment (T3). Participants were asked to answer all questions referring all time points. The survey took approximately 20 min to complete. All study procedures and materials were approved by the Institutional Review Board (IRB) of Rabin Medical Center (RMC 14-246).

### Measures

A standard questionnaire was used to collect demographic data (e.g., age, sex, race/ethnicity, education level, status of occupation and marital status). Disease-related variables, including cancer type, American Joint Committee on Cancer stage, treatment type (surgery alone, radiation, chemotherapy, or combined chemotherapy and radiation), whether participants were receiving treatment at the time of the study, and comorbid conditions were abstracted from the medical records. At the end of the survey, participants were able to provide any additional comments as free text. Key sentences that were used by the participants to describe the outcomes by free text at the end of the survey, were recorded as well (“Is there any comment you wish to add to the questions?”).

#### Health-related quality of life (HRQOL)

The 12-item Short-Form Health Survey (SF-12, version 2) is validated for use in adults older than 18 years and encompasses several domains as: general health and physical functioning, as well as social functioning, emotional limitations, and mental health [19]. Two overall subscales are derived that refer to physical and mental health components of quality of life. Cancer Rehabilitation Evaluation System (CARES) Sexual Functioning Summary Scale short form (SF), a validated tool to evaluate QOL issues and unmet needs among cancer patients [20–23] was used to assess sexual functioning. Participants are asked to assess sexual dysfunction, on a scale of zero (not at all) to 4 (very much). Scores represent the mean of ratings for each individual item and range from zero to 4. Unmet needs were measured using the Cancer Survivors’ Unmet Needs questionnaire (CaSUN) [24].

### Statistical analysis

Sample size was calculated to detect significant changes throughout time in the different modules, using professional assistance, yet we acknowledge the fact this is a small pilot study that may be underpowered (sample size was calculated to detect a minimal significant difference of 20 % (0.2) in at least one parameter yielding a sample size of *n* = 45.8 patients, with estimated drop out of 10 %). For HRQOL measures (symptoms) means were calculated for each time point for multivariate analysis using ANOVA with repeated measures test. For unmet needs, frequencies were calculated and analyzed per gender, stage and therapy using Fisher exact test. Multiple regression models were specified to examine associations between HRQOL outcomes and potential demographic (gender, age), cancer type, cancer stage (metastatic vs. local), treatment (single modality/ multimodality treatment) were added each to examine their impact. *P* < 0.05 was considered statistical significant. For the CARES scale, means were reported for categorical covariates (gender, cancer type) at each time point. Scores were compared between time points using ANOVA test. In order to gain a better understanding of how CRC patients may compare to other patient populations, standardized CARES scores were calculated. The female cohort was compared to breast cancer patients and non-breast cancer standardized norms [20] and the male cohort was compared to prostate cancer and non-prostate cancer standardized norms. All analyses were conducted in SPSS software.

## Results

### Participants

Fifty-three patients were eligible and approached to participate in the study. Fifty patients consented and completed the survey (94.3 % acceptance). Patient characteristics are presented in Table 1. Fifty-two percent were women. Median age at diagnosis was 35.5 years (range, 21–40 years old). Fifty percent of participants were diagnosed with colon cancer (*n* = 25), 30 % with rectal cancer (*n* = 15) and 8 % percent with gastric cancer (*n* = 4). Eighty percent were treated with multimodal treatments. The vast majority (94 %) had a full time job prior to their cancer diagnosis, and 62 % had higher education (college/university). Seventy four percent were married and 66 % had children at the time of diagnosis.

**Table 1:** Patient characteristics

Age	All n (%)	Female n (%)	Male n (%)
	50 (100)	26 (55 %)	24 (45 %)
	35.5 (20-49)	36	33
Cancer type			
Colon	25 (50 %)	14 (28 %)	11 (22 %)
Rectum	15 (30 %)	6 (12 %)	9 (18 %)
Gastric	4 (8 %)	3 (6 %)	1 (2 %)
Esophagus	2 (4 %)	1 (2 %)	1 (2 %)
Other	4 (8 %)	2 (4 %)	2 (4 %)
Stage at diagnosis			
I	4 (8 %)	1 (2 %)	3 (6 %)
II	12 (24 %)	6 (12 %)	6 (12 %)
III	19 (38 %)	11 (22 %)	8 (16 %)
IV	13 (26 %)	6 (12 %)	7 (14 %)
Unknown	2 (4 %)	2 (4 %)	
Treatment			
Surgery	7 (14 %)	3 (6 %)	4 (8 %)
Chemotherapy	3 (6 %)	1 (2 %)	2 (4 %)
Radiotherapy only			
Combined modality	40 (80 %)	22 (44 %)	18 (36 %)
Education pre diagnosis			
< 12 years of school	1 (2 %)		1 (2 %)
High school graduate	18 (36 %)	11 (22 %)	7 (14 %)
College/University	31 (62 %)	15 (30 %)	16 (32 %)
Employment status – pre-diagnosis			
Full time job	45 (90 %)	21 (42 %)	24 (48 %)
Part time job	2 (4 %)	2 (4 %)	
Unemployment	3 (6 %)	3 (6 %)	
Ethnicity			
Ashkenazi Jew	13 (26 %)	8 (16 %)	5 (10 %)
Sepheradi Jew	19 (38 %)	10 (20 %)	9 (18 %)
Mixed	5 (10 %)	3 (6 %)	2 (4 %)
Former USSR	8 (16 %)	2 (4 %)	6 (12 %)
Arab	2 (4 %)	2 (4 %)	
Other	1 (2 %)	1 (2 %)	
Marital status			
Married	37 (74 %)	19 (38 %)	18 (36 %)
Single	13 (26 %)	7 (14 %)	6 (12 %)
Married w/ children	33 (66 %)	13 (26 %)	20 (40 %)

### Symptom burden

**Fig. 1:**
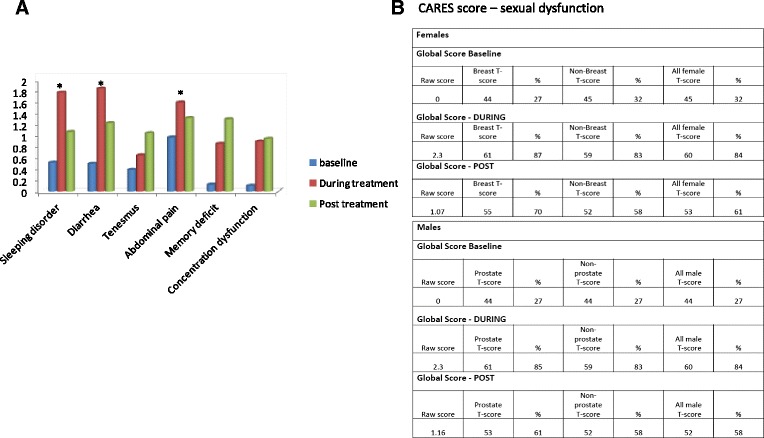
**a** Changes in symptom severity as reflected by the mean score (range 1-4). **b** Mean Cancer Rehabilitation Evaluation System (CARES) Sexual Dysfunction Scores by gender at different time points (baseline, during treatment and post-treatment). * represents *p* < 0.05

The symptoms that were significantly increased during and after treatments compared with baseline status were: diarrhea (37 % post treatment), sleeping disorder (32 % post treatment) abdominal pain (31 % post treatment); *p* < 0.05). The changes in symptom severity as reflected by the mean score are depicted in Fig. 1a. A substantial number of patients (40 %) reported they experience sexual dysfunction during treatment that improved post-treatment, but not to pre-treatment levels.

**Fig. 2:**
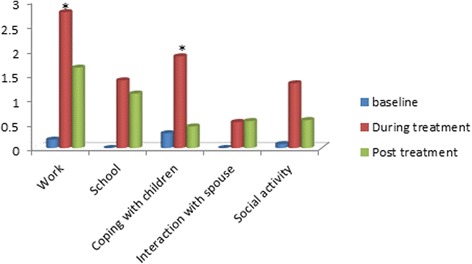
Changes in daily activities/social function as reflected by the mean score (range 1-4). * represents *p* < 0.05

The CARES reference scores were used to compare findings to normative samples in order to better characterize the similarities and differences of this group of YA patients/survivors with other patient populations. Using the CARES reference scores for breast cancer patients and for general female non-breast cancer patients, the raw score of female GI patients translated to the 85 and 87 percentiles during treatment and 70 and 58 percentiles post treatment, respectively. For male GI patients using the CARES reference scores for prostate cancer patients, general male non-prostate cancer patients, the raw score translated to the 85 and 83 percentiles during treatment and 61 and 58 percentiles post treatment, respectively.

In the psychosocial function domain, patients reported significantly lower scores referring to occupational activities and coping with children (*p* < 0.05) compared to pre-treatment scores as depicted in Fig. 2. Other parameters reflected marked difficulty during and post treatment but did not reach statistically significance. The observed pattern was similar in women and men, and not influenced by disease stage.

### Unmet needs

**Fig. 3:**
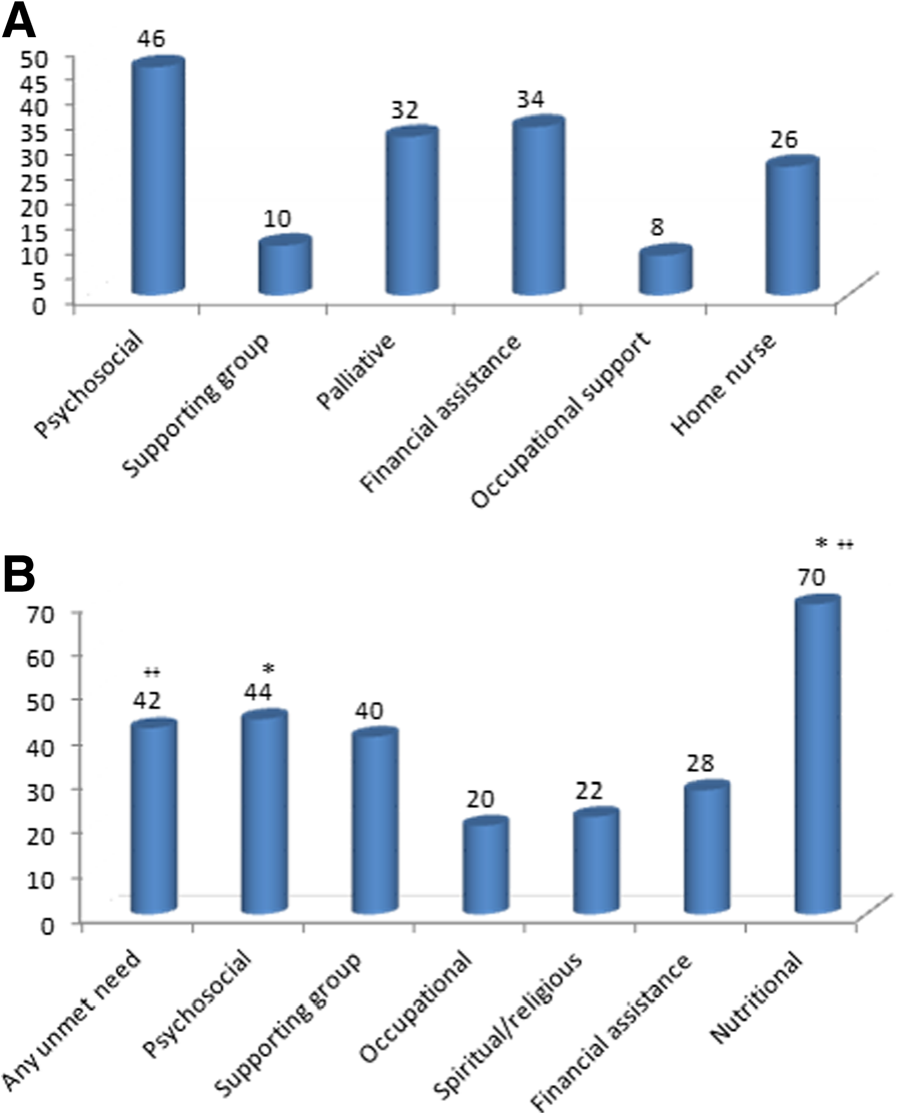
**a** Percentage of patient’s utilizing of supporting system. **b** Unmet needs analyzed by gender and treatment. (numbers represent %). * represents *p* < 0.05 for female versus male. ^++^ represents *p* < 0.05 for multimodality treatment versus single modality

Patients were asked to define the supporting systems they utilized in the medical institute. Psychosocial support, palliative support and financial counseling were the most prevalent services accessed using CaSUN (24; Fig. 3a). For evaluating the unmet needs domain, analyses compared the occurrence of an unmet need by gender, stage and type of therapy (Fig. 3b). Differences between unmet needs within the female cohort vs. the male cohort (more unmet need in women) were significant for nutritional counseling and psychosocial support (*p* < 0.05). Patients who were treated with multimodality treatment (surgery, chemotherapy and radiation) also presented higher rates of unmet needs (76 % vs 48 %; p-0.03).

## Discussion

This study assessed the reports and perceptions of YA with GI cancer regarding changes in their physical and psychosocial symptoms over time, as well as evaluating the emotional and practical needs during treatment and throughout early survivorship.

The results indicate that following treatment young GI cancer patients experience symptoms that significantly worsen their QOL and interfere with daily activities as diarrhea, sleeping disorder and sexual dysfunction. In the psychosocial function domain, patients reported significant impairment in occupational activities and coping with children.

The psychosocial aspects young adult cancer patients experience are gaining increased focus in recent studies [25–28], including consequences for QOL indicators (e.g., interpersonal relationships with spouse/family, coping with young children, relative disability to conduct daily activities, fertility issues) [29]. Our study indicates that young adults with GI cancers experience cancer- and treatment-related symptoms in a pattern that resembles the impact of cancer on AYA patients as appear in former studies [26], which showed that having unmet service needs was strongly associated with lower health related quality of life. In former studies, a large proportion of AYAs pointed at an unmet need for counseling mainly for physical exercise and nutrition, thereby reflecting a gap in obtaining supporting services. Recent study sought to explore the needs and preferences of colorectal cancer survivors (in all ages), and found that the respondents to the study survey reported receiving more medical information about their cancer or its follow-up than about nonmedical issues, such as support groups, counseling, and financial and insurance issues. The authors concluded that the lack of communication regarding nonmedical issues highlights the need for multidisciplinary support for colorectal cancer survivors [30]. In a study that compared the symptom burden of young patients with breast or colorectal cancer compared with older counterparts, the young patients experienced a worse symptom burden, especially in colorectal patients [6].

Our patients reported significant unmet needs for nutritional counseling and psychosocial support mainly among women. Other needs as occupational counseling, spiritual support, financial counseling and palliative support were documented as well yet, not in a specific gender. Patients who were treated with multimodality treatment (surgery, chemotherapy and radiation) presented higher rates of unmet needs. We found no differences in unmet needs between metastatic setting and adjuvant setting, or between younger ages (<35 years at diagnosis). Female patients depicted a trend towards higher rate of unmet needs. We used the CARES- SF score, an instrumental for assessing the unmet needs of patients with cancer in a standardized method providing a normative to specific cohorts of cancer patients [20, 22] suited for patients anywhere along the cancer continuum that was shown to be especially useful for evaluating the rehabilitation needs of survivors of cancer. The CARES score enabled us to compare our cohort to breast cancer patients and general non-breast cancer female patient population (for the female patients) and prostate cancer and general non-prostate cancer male patient population (for male patients): At baseline both female and male GI patients had no symptomatic sexual symptoms. During the treatment, the degree of sexual dysfunction experienced by the patients resembled high percentile of breast cancer patients (equivalent to 87^th^ percentile) and of general female patient population (84^th^ percentile). As for the male GI patients – compared with prostate cancer patients the degree of sexual dysfunction was averaged (59^th^ percentile), but markedly high compared with general male patient population (83th percentile). We acknowledge that these scales are not established specifically for young patients, but have been adopted in other studies of characterizing young-onset population [31], and moreover, that younger population may have a better starting point that older patients with regard to sexual function, mainly due to lack of menopausal symptoms that may exacerbate sexual dysfunction. Sexual dysfunction in young breast cancer patients has been formerly documented [31] and correlated to treatment-related amenorrhea. In our cohort, the majority of patients remained menstruating throughout treatment or resumed menses shortly after treatment cessation (for colon cancer patients). Two rectal cancer patients had amenorrhea following chemoradiation. According to free text answers the patients provided regarding sexual function – there was loss of interest mainly due to diarrhea, abdominal pain and increased peristalsis, and general transient “loss of body image”. For patients who were treated with chemoradiation, following radiation sexual dysfunction CARES scores were higher than the colon cancer or gastric cancer patients (data not shown). Our study highlights the need for potential intervention regarding vaginal dilators for rectal cancer patients, and psychological counseling for others. For patients that experience menopausal symptoms during treatment-induced amenorrhea, early intervention with topical estrogenic creams may be useful to alleviate vaginal atrophy and discomfort. A recent study evaluated the relations between body image and sexual function in a cohort of female patients with rectal and anal cancer. The median age of the participants was 55 years. About half of the cohort endorsed two or more body image problems, and 28 % described feeling quite a bit or very much concerned about at least one problem. Younger age, lower global health status, and worse gastrointestinal tract symptoms, in particular, were related to poorer body image [17]. An interesting observation reflected from our study is that despite the significant deterioration in sexual dysfunction, it does not translated into interrupted interaction with the spouse, whose score remains relatively stable throughout the time-points.

A major issue that was reflected as a substantial unmet need was nutritional counseling, as reported by 70 % of the participants. Suboptimal absorption and lack of adequate nutrition in patients treated for colorectal cancer as well gastric cancer have been documented in several studies. Former evidence indicate that early individualized nutritional counseling and education of cancer patients treated with radiotherapy is highly effective [32]. Nutritional factors are implicated in the different milieu of anti-cancer therapies (surgery, chemotherapy, radiotherapy and multi-modality treatments), as well as tumor-related consequences and psychosocial factors as depression and anxiety [33–35]. Unintentional weight loss and malnutrition may occur in 30 to 80 % of cancer patients, while patients with GI cancers are considered highly prone to develop these symptoms. Recently Platek et al [36] conducted a cross-sectional Study that aimed to determine the prevalence and types of outpatient clinical nutrition services available at comprehensive cancer centers (CCCs). The authors concluded that CCCs rely on referral-based clinical nutrition service, which are not consistently a part of multidisciplinary care, and that an in-depth comparison of clinical nutrition services among other approaches to cancer care, including a comparison of clinical outcomes among these different approaches, is needed.

Our study carries several limitations. Patients were enrolled to the study following the cessation of treatment, acknowledged as “cancer survivors”, and hence baseline values may be subjected to recall bias. Furthermore, the sample is comparatively small and heterogeneous (several tumor types) mainly due to the relatively low prevalence of GI cancers in young adults who are less than 40 years that were accessible for enrollment at the time of the study. Nonetheless, the results show clear trend of specific symptoms and unmet needs that hamper the QOL of these young adults and warrant consideration of the medical-psychosocial aspects. The purpose of the study had been to pave the ground for a large prospective study which is ongoing in our center, in which all newly diagnosed young patients with GI cancers are enrolled and assessment is performed longitudinally, in a timely fashioned. Future prospective large scale studies are required to delineate the magnitude of unmet needs as well as physical and psychosocial symptoms in a prospective fashion, to better tailor suitable interventions, and to further compare young patients with GI cancer to other young-onset cancer patient cohorts.

## Conclusion

In conclusion, the current study suggests that young patients with GI cancers represent a group with unique characteristics and needs compared with published evidence on other young-onset malignancies. The distinctive symptoms and treatment-related functional impairments indicate there are unmet needs, especially in the area of psychosocial support and nutritional counseling. In-depth studies are necessary to determine the availability and access to these service needs, and which subsets of individuals can benefit them the most.

## Abbreviations

CRC: Colorectal Cancer
GI: Gastrointestinal
HRQOL: Health Related Quality of Life
QOL: Quality of Life
YA: Young Adults
